# Microbial Melanin-Reinforced Chitosan Films with Enhanced Functional Performances

**DOI:** 10.1021/acsomega.6c06855

**Published:** 2026-08-26

**Authors:** Talayeh Kordjazi, Marika Avitabile, Teresa Gervasi, Massimo Rippa, Teresa Paduano, Simona Zuppolini, C. Valeria L. Giosafatto, Loredana Mariniello, Odile Francesca Restaino

**Affiliations:** † Department of Chemical Sciences, 98793 University of Naples Federico II, Monte Sant’Angelo Campus, via Cintia 4, Naples 80126, Italy; ‡ Department of Biomedical and Dental Sciences and Morphofunctional Imaging, 18980 University of Messina, Viale Annunziata 38, Messina 98122, Italy; § 96973 Institute of Applied Sciences and Intelligent Systems “E. Caianiello” of CNR, Via Campi Flegrei 34, Pozzuoli 80078, Italy; ∥ Institute for Polymers, Composites and Biomaterials of CNR, P.le E. Fermi 1, Portici, Naples 80055, Italy

## Abstract

Melanin is a polymeric pigment with numerous properties like UV–visible light protection, and antioxidant and antimicrobial activities. In this study, for the first time, a microbial melanin, eumelanin-like, and biotechnologically produced by *Streptomyces nashvillensis* DSM 40314, was incorporated in chitosan films to develop multifunctional biobased materials. A4 films containing 1.0–4.0% (w/w) melanin were prepared by the casting method and then characterized for their physicochemical, mechanical, and biological properties, in comparison with control films. FT-IR analyses confirmed interactions between chitosan and melanin, and the films showed improved mechanical properties, such as enhanced tensile strength and flexibility. Water solubility of the films decreased with pigment insertion, while moisture content and swelling increased, suggesting changes in the polymer network. UV–visible shielding and gas barrier properties were also significantly enhanced. Films containing 4.0% melanin exhibited strong antioxidant activity (88.34 ± 0.02 μM FRAP; 49.22 ± 5.60% DPPH) and antimicrobial activity against*P. aeruginosa* ATCC 27853 and *Enterococcus faecalis* ATCC 29212, compared to the control. Overall, microbial melanin acted as a multifunctional bioactive reinforcement in chitosan-based films, improving their functional performances useful for potential applications in food packaging and biomedical fields.

## Graphical abstract

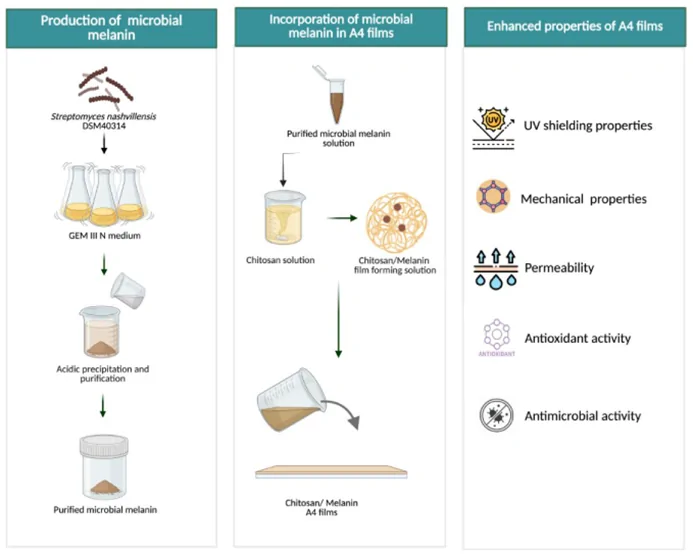

## 1 Introduction

The necessity to overcome the production and use of petroleum-based materials made of synthetic polymers has been growing more and more in the last three decades, also due to restricted international laws that support more sustainable and ecological industrial processes.^1^ To avoid nonbiodegradable plastic products and reduce their impact on the environment, scientific research has developed new, alternative, biobased materials by using naturally derived polymers, like carbohydrate or protein polymers, which have the advantage of being renewable, biodegradable, biocompatible, and mostly nontoxic. Besides, the insertion of bioactive molecules in these films, like antimicrobial agents, antioxidants, or pigments, has opened the possibility to potentially increase or modify the initial physicochemical, mechanical, and barrier properties of the materials and to drive toward tailored characteristics.^2,3^ Chitosan is a natural, linear polysaccharide made of N-acetyl-D-glucosamine units, partially deacetylated; it is very abundant in nature, being derived from chitin present in marine crustacean organisms.^4^ In recent decades, chitosan has gained significant scientific and industrial interest thanks to its good film-making ability and mechanical properties, edibility, biocompatibility, biodegradability, nontoxicity, water retention capacity, a good oxygen barrier, and antimicrobial properties. All these characteristics have made it useful in the manufacturing of sustainable, biodegradable plastics for food packaging, edible films, protective layers for drug delivery systems, skin cosmetic formulations, and advanced therapeutic biomaterials as components of wound dressings, tissue engineering scaffolds, and surgical implants.^5–7^ Besides, different strategies have also been explored to incorporate nanoparticles or bioactive molecules into chitosan-based films or materials to add specific properties, with the perspective to expand the possible range of applications.^8^ For example, in the biomedical field, numerous chitosan-based nanocomposite films have been prepared for wound-dressing applications having antimicrobial properties thanks to the release of bioactive compounds such as silver nanoparticles, antibiotics, or growth factors.^9^ Among the biomolecules that might be inserted in chitosan materials and/or films, pigments—and in particular melanin—have been very little explored so far. Melanin is a pigment widely found in all kingdoms—animal, plant, and microbial worlds—whose colors might vary from reddish to brown to dark brown, according to its structure.^1,2,4^ This pigment has peculiar physicochemical properties like high thermal stability, good UV–visible light absorption capabilities, metal ion-chelating ability, hybrid ionic-electronic conductance, and redox properties that make it suitable for numerous industrial applications in food packaging, cosmetics, green (bio)­electronics, and water decontamination.^6,9^ Besides, melanin also shows antioxidant and antimicrobial properties, biocompatibility, biostability, no cytotoxicity, and no antigenic response, so its incorporation in chitosan films might enhance some features of the polysaccharide polymer (e.g., antimicrobial) and also add new specific properties like UV–visible light absorption abilities, thermal stability, and antioxidant activity. So far, in the literature, only animal-derived or synthetic melanin has been used for the preparation of chitosan-based materials,^10,11^ but its use has been limited by the high costs of recovery and purification from tissues in the case of animal-derived melanin (e.g., from the ink of the cuttlefish *Sepia officinalis*) or of the synthetic routes required to obtain the chemical-derived pigment.^12,13^ Recently, biotechnological production of microbial melanin has been explored as an alternative, more sustainable route of pigment manufacturing compared to the animal extraction or synthetic production process.^13^ In particular, some bacterial species have been employed as microbial cell factories to produce this pigment,^13,14^ like *Streptomyces nashvillensis* DSM 40314, which has already been demonstrated to produce a eumelanin-like pigment, synthesized and released as an extracellular product during secondary metabolism. The yield of microbial melanin production might be boosted by applying different fermentation strategies and by modulating growth conditions and medium composition.^13,15–17^ To the best of our knowledge, microbial melanin has never been inserted in any chitosan films or materials, nor in the form of a dispersed powder. In this paper, for the first time, the microbial melanin produced by *S. nashvillensis* DSM 40314 on a glucose, yeast extract, and malt extract medium, and then purified, was inserted into chitosan-based films. The films were then characterized to evaluate the effect of microbial melanin incorporation on their performance compared to a control. A comprehensive set of analyses was employed to determine changes in structural organization, physicochemical stability, and functional behavior. In particular, the evaluation of optical, mechanical, barrier, and biological properties was performed, providing critical insight into the suitability of these materials for advanced applications in the future.

## 2 Materials and Methods

### 2.1 Materials

For melanin biotechnological production, medium nutrients like yeast and malt extracts were from Himedia Laboratories (India), while glucose, NaH_2_PO_4_·H_2_O, Na_2_HPO_4_, and HCl used for melanin purification were from Sigma-Aldrich (USA). *Streptomyces nashvillensis* DSM 40314 was purchased from DSMZ (Germany). Methanol, 2,2-diphenyl-1-picrylhydrazyl (DPPH), and the FRAP assay kit (MAK509) were purchased from Sigma-Aldrich (USA). Acetic acid was purchased from Merck (Germany), and glycerol by Carlo Erba (Milan, Italy). Chitosan (molecular weight 184 kDa, degree of deacetylation ≥ 90%, viscosity 50 to 200 mPa·s; Thermo Scientific, USA) was used as the polysaccharide matrix for film preparation,

### 2.2 Melanin Biotechnological Production and Purification

Melanin was produced by *Streptomyces nashvillensis* DSM 40314 in 1-L shake flasks on GEM III N medium [glucose (12.0 g·L^–1^), yeast extract (6.0 g·L^–1^), malt extract (30.0 g·L^–1^), NaH_2_PO_4_·H_2_O (5.8 g·L^–1^), Na_2_HPO_4_ (8.2 g·L^–1^)] at pH 7.0, 28 °C, and 250 rpm in a rotary air shaker (ISF-1-W, Kühner, Switzerland), according to previously reported protocols.^14,15^ After 96 h, the runs were stopped, and the broths were centrifuged at 4 °C, 5000 rpm for 20 min (Avanti J-20XPI, Beckman Coulter, USA); the cellular biomasses were discarded, and separated by the clarified broth supernatants. Melanin was precipitated by addition to the supernatant volumes of 5.0 M HCl up to pH 1.5 and recovered after overnight incubation at 4 °C by centrifugation at 4 °C and 5000 rpm for 20 min (Avanti J-20XPI, Beckman Coulter, USA).^17^ The precipitated melanin was then purified according to a previously reported protocol^15–17^ by performing several washes, in series, with 5.0 M HCl first, and then with MilliQ water, at room temperature for 24 h. The pigment was then recovered by centrifugation at 4 °C and 5000 rpm for 20 min (Avanti J-20XPI, Beckman Coulter, USA) and dried at room temperature. The final melanin concentration obtained in the shake flask runs was checked by measuring the absorbance at 220 nm of the clarified broth supernatant (Spectrophotometer V-530, Jasco, Japan), according to previously reported protocols.^14,15^ In addition, the purity of the pigment, in percentage, was determined by measuring the absorbance at 220 nm (Spectrophotometer V-530, Jasco, Japan) after dissolving a precise amount of the dried material in a defined volume of 0.1 M NaOH, also used as the blank, and by dividing the obtained concentration with the theoretical concentration value of the weighed dried material.^15,16^ The dried, purified melanin was then inserted into the chitosan-based films. The microbial melanin used in this study was characterized similarly to previously reported studies by UV–visible and FT-IR analyses (Restaino et al., 2024).

### 2.3 Preparation of Chitosan Films

Chitosan-based films incorporating various concentrations of microbial melanin were prepared in duplicate by using the casting method.^18^ To prepare the film-forming solutions (FFSs), 2.6 g of chitosan powder were dissolved in 100 mL of 1.0% (v/v) acetic acid by heating at 90 °C for 30 min under vigorous stirring. The purified melanin powder was dissolved at different percentages (1.0, 2.0, 3.0, or 4.0% w/w, on the base of chitosan weight) in 1.0 mL of MilliQ water and then added to the chitosan solution, followed by the incorporation of 30.0% (w/w, on the base of chitosan weight) of glycerol as a plasticizer. The FFSs were stirred for an additional 30 min and then cooled to room temperature. The effect of melanin incorporation into FFSs was evaluated by measuring zeta potential and particle size distribution. Zeta potential measurements were carried out at 25 °C in triplicate using a Zetasizer Nano-ZSP (Malvern, Worcestershire, UK) and calculated from electrophoretic mobility by using the Henry equation (Zetasizer software v7.12). For both zeta potential and particle size measurements, samples were diluted to a final concentration of 1.0 mg/mL in MilliQ water. Particle size and the polydispersity index (PDI) were determined by dynamic light scattering. The FFSs were cast by using a coat master (Coatmaster 510, Erichsen, Iserlohn, Germany) in an A4-like area (210 × 297 mm). The films were dried at room temperature for 24 h after detachment from the casting surface, and then subjected to characterization. The same procedure was used for the film as control, without incorporating melanin.

### 2.4 Characterization of the Films

#### 2.4.1 Scanning Electron Microscopy Analyses

The surfaces and cross-sections of all chitosan-melanin films and the control were analyzed in duplicate by using scanning electron microscopy (SEM). Each sample was placed on a support covered with a carbon adhesive film. Before measurement, all samples were coated with gold-palladium alloys by using a vacuum sputtering coater (Denton Vacuum DeskV) and then observed with a field-emission scanning electron microscope (Nova NanoSEM 450, FEI Instruments) at an accelerating voltage of 3 kV, with an Everhart–Thornley detector and a through-the-lens detector for the magnifications.^19^

#### 2.4.2 Fourier Transform Infrared Spectroscopy

Fourier-transform infrared (FT-IR) analyses of all chitosan films added with melanin and the control were recorded twice by using an FT-IR-4700 instrument (JASCO, Japan) in the range of 4000–400 cm^–1^, at room temperature.^20^

#### 2.4.3 Colorimetric Analysis of the Surface, UV-Shielding, and Optical Properties

Colorimetric analyses were performed in triplicate on the four chitosan films incorporating different melanin concentrations and on the control films. Measurements were conducted in a pump–probe configuration, assessing light absorption under diffuse reflectance conditions by using a spectrophotometer (MAYA2000Pro, Ocean Insight, Oxford, UK). The illumination was provided by a halogen lamp corresponding to the CIE D65 standard illuminant, with an emission range between 400 and 1000 nm. Prior to data acquisition, the instrument was calibrated using a white ceramic reference tile and a black trap. Spectral reflectance, colorimetric parameters, and basic processing were obtained through the OceanView 2.0 software (Ocean Insight, Oxford, UK) supplied with the device. For each film, the probe was gently placed in contact with the surface, and ten measurements were taken at different locations across the sample. The average colorimetric values for each specimen were then calculated by evaluating the dominant wavelength (λDOM) as well as the corresponding x and y chromaticity coordinates in the CIE diagram 1931. To check the UV–visible shielding capacities of the chitosan films, without or with melanin, transmittance spectra were run in triplicate by using a spectrophotometer (Spectrophotometer V-530, Jasco, Tokyo, Japan) in the wavelength range of 200–800 nm. Film opacity, instead, was calculated on the basis of the film absorbance, measured in triplicate at 600 nm, by using the following equation:

1 $$\mathrm{Opacity}=\frac{\mathrm{Abs}600}{t}$$

where Abs600 was the absorbance at 600 nm and *t* was the thickness of the film (mm).^21^

#### 2.4.4 Thermo-Gravimetric Analyses

Thermo-gravimetric analyses (TGA) of all films added with microbial melanin and of the control were performed in duplicate by using a Netzsch TG 29 F1 Libra system (Netzsch Geratebau GmbH, Selb, Germany) within the temperature range 25–800 °C. The analyses were conducted under N_2_ atmosphere (50 mL/min) with a heating ramp of 10 °C/min. Samples having weights of around 5.0 ± 0.5 mg were used for the run tests.^22^

#### 2.4.5 Thickness and Mechanical Properties

The thickness of all melanin–chitosan-based films and the control were measured by using a hand-held digital micrometer (IP65, ALPA Metrology, Italy) at five random points, and then an average value was calculated. Mechanical properties, including tensile strength (TS) and elongation at break (EAB), were determined by using a universal testing machine (Model 5543A, Instron Engineering Corp., Canton, MA, USA). Before measurements, films were precisely cut into rectangular strips (1 cm × 4 cm) and conditioned at 25 °C and 50.0% relative humidity (RH) for 72 h to reach equilibrium. The instrument was adjusted to stretch mode using a 200 N load cell; the initial grip separation and the crosshead speed were set to 50 mm and 50 mm/min, respectively. TS (MPa) was determined by dividing the maximum load at film break by the initial cross-sectional area of the film sample. EAB (%) was determined by the percentage of elongation at break of the films to the initial length of the films. At least ten rectangular strips were precisely cut for each type of sample.^19^

#### 2.4.6 Moisture Content, Water Solubility, and Swelling Ratio

The effect of melanin incorporation on the water-related properties of all chitosan films was evaluated and compared to the control by measuring the moisture content (MC), water solubility (WS), and swelling ratio (SR). All measurements were performed in triplicate using film samples measuring 2 cm × 2 cm. The moisture content of the films was determined gravimetrically as mass loss (gravimetric moisture content): the initial mass of each film sample (M1) was recorded, then the samples were dried in a hot-air oven at 105 °C for 24 h, according to the ASTM method (ASTM E96/E96M-24a), then cooled to room temperature in a desiccator of MgNO_3_ to minimize moisture reabsorption, and weighed to determine their dry mass (M2). The moisture content percentage was calculated by using the following equation:

2 $$\mathrm{MC}\left(\%\right)=\frac{M1-M2}{M1}\times 100$$

where M1 was the initial weight (mg) of each film sample and M2 (mg) was the dry weight of each film sample.

To measure the water solubility (WS), samples for each film (2 cm × 2 cm), in triplicate, were dried at 105 °C for 24 h and the initial dry weight was determined. Then the samples were cooled at room temperature in a desiccator before being weighed and the resulting initial dry mass was recorded as M2. The dried samples were then immersed in 50 mL of distilled water and gently stirred at 25 °C for 24 h. After immersion, the remaining insoluble film pieces were recovered and dried again at 105 °C for 24 h. The samples were cooled in a desiccator and weighed to determine the final dry mass, recorded as M3. Water solubility was then calculated according to the following equation:

3 $$\mathrm{WS}\left(\%\right)=\frac{M2-M3}{M2}\times 100$$

where M2 was the initial dry weight of the film (mg), and M3 (mg) was the final dry weight of the film.

To calculate the swelling ratio (SR), all film samples (2 cm × 2 cm) and the control, in triplicate, were first weighed and then soaked in 50 mL of distilled water for 24 h. After taking the film samples out of the distilled water, residual water on their surfaces was completely removed with a piece of filter paper, and their final weights were determined. The SR of all the films was calculated according to the following formula:

4 $$\mathrm{SR}\left(\%\right)=\frac{M1-M4}{M1}\times 100$$

where M1 was the initial weight (mg) and M4 (mg) was the final weight of the films after swelling.

#### 2.4.7 Gas Permeability

The carbon dioxide (CO_2_) and water vapor permeability (WVP) of both melanin films and the control samples (2 cm^2^) were determined in triplicate by using a MultiPerm analyzer (ExtraSolutions s.r.l., Pisa, Italy) according to modified ASTM protocols.^18^ Before being tested, all films were equilibrated for 24 h at 50.0% relative humidity (RH).

#### 2.4.8 Water Contact Angle

The surface hydrophobicity of the films was evaluated by measuring the water contact angle (WCA). Measurements were performed by using a continuous-zoom camera (Jiosion, China). Films were neatly cut and fixed onto a flat surface. Five droplets of distilled water (10 μL each) were deposited at different locations on both sides of each film, and the contact angle was calculated using ImageJ software (version 1.53t). The reported WCA values represent the mean of these measurements.^23^

#### 2.4.9 Antioxidant Activity

The ferric reducing antioxidant power (FRAP) assay was performed by using the MAK509 kit according to the manufacturing procedure (Sigma-Aldrich, USA). Briefly, 10 mg of dried films were dissolved in 1 mL of distilled water to obtain the film extracts. Aliquots of 50 μL of each extract were mixed with 200 μL of FRAP working solution in a 96-well microplate. The plate was then incubated at room temperature for 40 min, after which the absorbance was measured at 590 nm using a microplate reader (FlexA-200 monochromator microplate reader; France). A standard curve was generated using ferrous sulfate (Fe^2+^) standards ranging from 0 to 180 μM. The FRAP values were expressed as μM Fe^3 +^ equivalents according to the following equation:

5 $$\left[\mathrm{FRAP}\right]{(\mu \mathrm{M Fe}}^{2+}\mathrm{equivalents})=\frac{R\mathrm{sample}-R\;\mathrm{Blank}}{\mathrm{Slope}}\times \mathrm{dilution factor}$$

The antioxidant activity of chitosan-based films was also evaluated by using the DPPH (1,1-diphenyl-2-picrylhydrazyl) according to previously reported methods^24^ with slight modifications: 10 mg of each film was dissolved in 1 mL of a 1.0% acetic acid solution. For each assay, 100 μL of a 67.5 μmol/L solution of DPPH in methanol was mixed with 10 μL of the different sample solutions and 190 μL of methanol in a 96-well microplate, and the assays were performed in triplicate. The reaction mixtures were incubated for 1 h at room temperature in dark conditions and then the absorbance was measured at 517 nm by using a microplate reader (FlexA-200 monochromator microplate reader, France). For the control sample, 10 μL of a 1.0% acetic acid solution was used in replacement of the film solutions, while for the blank sample only methanol (290 μL) and 10 μL of a 1.0% acetic acid solution were mixed. The percentage inhibition of radical-scavenging activity was calculated using the following equation:

6 I(%)=[(Ac −
As)/Ac]×100

where Ac was the absorbance of the control and As was the absorbance of the sample.

#### 2.4.10 Antimicrobial Activity

Tests were carried out to determine the antibacterial activity of the best chitosan-based FFS against *E. faecalis* ATCC 29212 and *P. aeruginosa* ATCC 27853 by using a broth microdilution assay for bacteria based on colony count methods.^25^ FFS functionalized with 4.0% melanin was added to Mueller-Hinton broth (MHB, Oxoid, Sigma, Italy) (300 μL) containing a standardized bacterial load at a concentration of 5 × 10^5^ CFU•mL^–1^. Bacterial growth was evaluated after 24 h at 37 °C by plating serial dilutions. Plates were incubated at 37 °C for 24–48 h, and the number of colonies was reported as CFU•mL^–1^. Control FFS without melanin was also tested.

### 2.5 Data and Statistical Analyses

The data reported in this paper were the average values of different independent experiments. Statistical analysis was performed by using GraphPad Prism (version 10.0, GraphPad Software, USA). The normality of data distribution was assessed using the Shapiro–Wilk test, and the homogeneity of variances was evaluated using the Brown–Forsythe test. Data were expressed as mean values with standard deviations. One-way analysis of variance (ANOVA), followed by Tukey’s multiple comparison test, was applied to evaluate differences among groups. Differences were considered statistically significant at *p* < 0.05.

## 3 Results

### 3.1 Preparation of Films Added with Microbial Melanin

The final concentration of melanin, obtained in shake flasks on GEM III N medium by*Streptomyces nashvillensis* DSM 4031, was similar to the value already reported in previous papers (0.75 ± 0.01 g•L^–1^),^15^ while the purity reached values of up to 95.0 ± 0.1%. The obtained melanin was confirmed to be an eumelanin-like pigment based on its characteristic broadband UV–visible absorption profile and FT-IR spectral features (data not shown), as previously reported (Restaino et al., 2024).^16^ The purified microbial melanin was then used in chitosan FFSs as an additive at different percentages (Figure 1A). The results of zeta potential of FFSs showed a progressive increase in Z-size from 1418.0 ± 44.2 (pure chitosan) up to 2969.0 ± 20.5 nm for the film with 4.0% melanin (Table 1) after pigment incorporation. The increase in particle size suggested a more extended polymeric assembly in aqueous suspension. Zeta potential increased from + 33.8 ± 0.29 mV to + 69.1 ± 0.70 mV with increasing melanin content, indicating an enhanced surface charge density and improved colloidal stability. PDI decreased from 0.847 ± 0.02 to 0.513 ± 0.01, also suggesting a more homogeneous particle size distribution by increasing microbial melanin content. These findings suggested strong intermolecular interactions between chitosan and melanin by electrostatic attractions and hydrogen bonding (Table 1). From the FFS solutions, A4 films were prepared by the casting method (Figure 1B) and they showed very homogeneous structures in which the pigment was totally dispersed. The neat chitosan film was freestanding, transparent, and colorless, while the melanin-containing films remained transparent but showed a brown color that became darker as the concentration of melanin raised.

**Figure 1:**
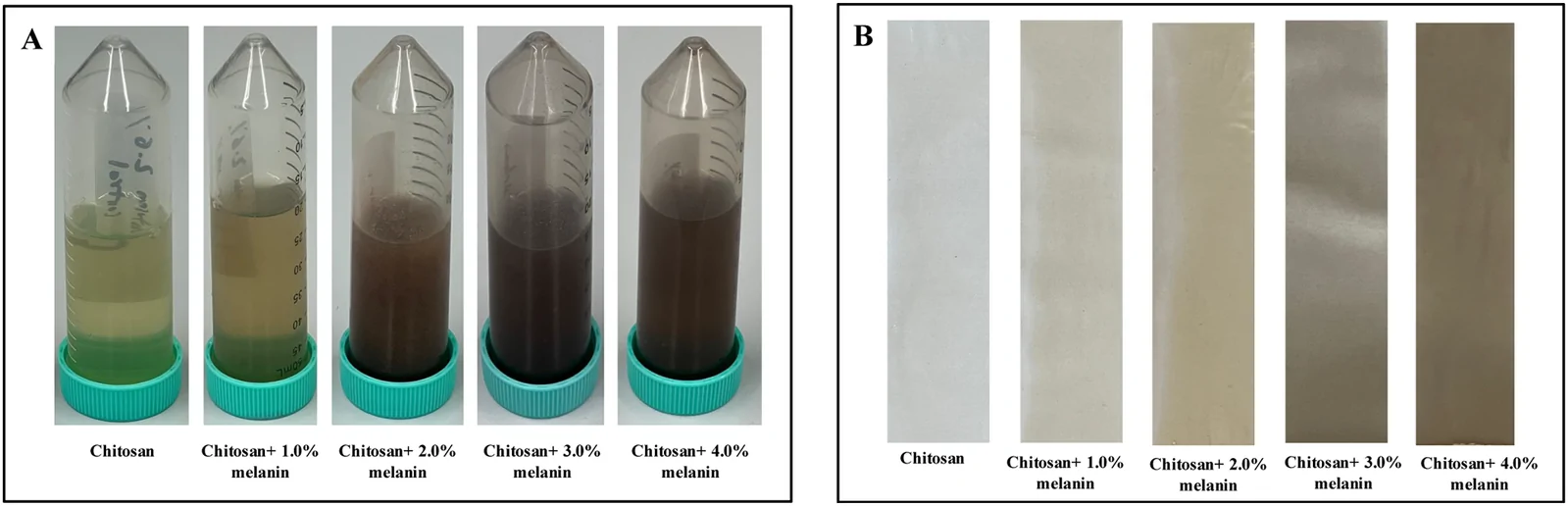
Different FFSs (A) of chitosan, used as a control, and with the addition of different percentages (from 1.0 to 4.0%) of microbial melanin, and the corresponding manufactured films (B).

**Table 1:** Size (Z-Size), Surface charge potential (Z-Potential) and polydispersity index (PDI) of the different chitosan FFSs with and without microbial melanin^a^

	Z-Size (d.nm)	Z-Potential (mV)	PDI
**Chitosan**	1418 ± 44.18^e^	+33.8 ± 0.29^d^	0.847 ± 0.02^a^
Chitosan+ 1.0% melanin	1839 ± 86.19^d^	+52.6 ± 2.28^c^	0.668 ± 0.04^b^
Chitosan+ 2.0% melanin	2261 ± 17.09^c^	+56.6 ± 2.25^c^	0,626 ± 0.0^b^
Chitosan+ 3.0% melanin	2813 ± 61.78^b^	+62.6± 0.81^b^	0.543 ± 0.04^c^
Chitosan+ 4.0% melanin	2969± 20.50^a^	+69.1 ± 0.70^a^	0.513 ± 0.01^c^

a Data are presented as average values with standard deviations. Different letters within the same column indicate significant differences (p < 0.05),

### 3.2 Characterization of the Films

#### 3.2.1 Scanning Electron Microscopy Analyses

Scanning electron microscopy analyses of the surfaces and cross-sections of all chitosan films containing microbial melanin and the control were performed (Figure 2A and B). The neat chitosan film exhibited a relatively smooth and homogeneous surface, with minor microcracks probably associated just with the drying process. The cross-sectional analysis revealed a dense and compact internal structure with a layered morphology typical of solvent-cast films. Upon incorporation of 1.0% melanin, the film maintained a continuous and compact structure, and the surface showed a slight increase in roughness compared to the control, while the cross-section remained dense and layered. Adding 2.0%, 3.0%, and 4.0% microbial melanin, surface irregularities became more noticeable, and textural heterogeneity appeared. Correspondingly, the cross-sectional images showed internal structural variations with reduced uniformity compared to the control.

**Figure 2:**
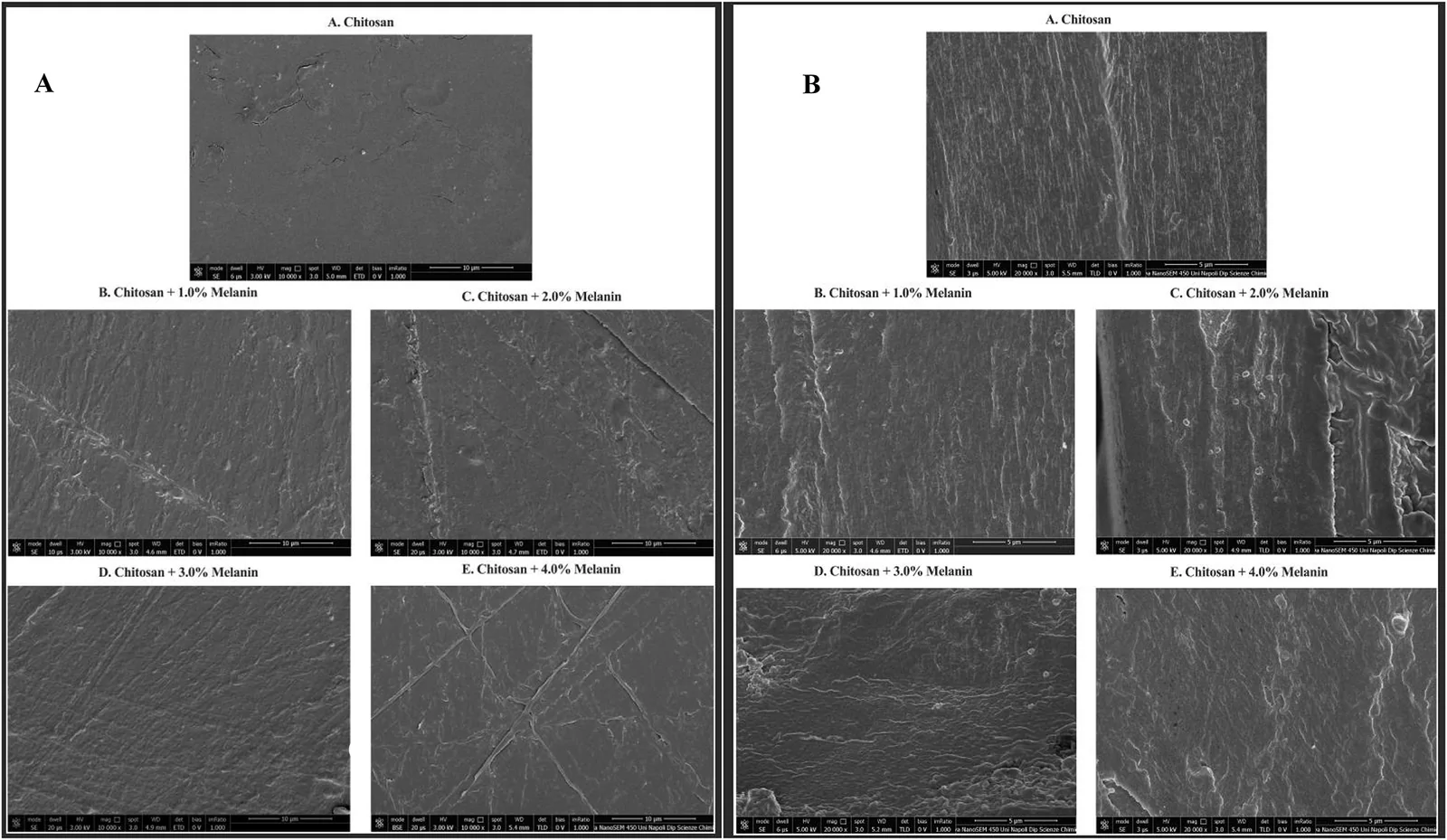
Surface (A) and cross-section (B) images of the chitosan film as a control (A), and of the chitosan films added with 1.0% (B), 2.0% (C), 3.0% (D), and 4.0% (E) microbial melanin.

#### 3.2.2 Fourier Transform Infrared Spectroscopy Analyses

Fourier-transform infrared spectroscopy (FT-IR) analyses of the chitosan films, as a control, and of films added with different percentages of microbial melanin provided insights into the interactions between chitosan and the pigment (Figure 3). All the samples exhibited a broad band around 3200–3400 cm^–1^, attributed to O–H and N–H stretching vibrations, which is a characteristic feature of chitosan. This peak became slightly broader and less intense with the increase of melanin content, indicating possible interactions between the amino groups of chitosan and the phenolic groups of melanin.^10,26^ The peak at around 2920 cm^–1^, corresponding to C–H stretching, remained unchanged, suggesting that the aliphatic backbone of chitosan was not involved in the interactions with melanin.^11^ The peaks at about 1650 cm^–1^ and 1590 cm^–1^, corresponding to the amide groups, exhibited shifts and intensity changes in the melanin chitosan-based films, confirming the interactions between chitosan and melanin.^26^ Additionally, peaks in the region of 1400–1250 cm^–1^, associated with C–H bending and C–N stretching of chitosan, showed enhanced intensities when the microbial melanin was incorporated in the films, probably due to the aromatic nature and the nitrogen-containing structures of melanin.^27^ The C–O–C asymmetric stretching peak at ∼1150 cm^–1^ and the C–O stretching vibrations between 1070–1020 cm^–1^ also showed slight modifications, suggesting that the chitosan backbone underwent minor structural rearrangements due to interactions with the pigment.^21^ Overall, the absence of new peaks in the spectra compared to the control, with only changes in peak intensities, confirmed charge interactions between the polymer and the pigment. These FT-IR data were consistent with the colloidal data where melanin addition produced larger and more homogeneous aggregates (Z-size increases with decreasing PDI) and an increase in z-potential, indicating enhanced surface charge density and electrostatic stabilization. FTIR, z-potential, size, and PDI results support the formation of supramolecular chitosan–melanin complexes primarily mediated by hydrogen bonding and electrostatic interactions rather than simple physical mixing.

**Figure 3:**
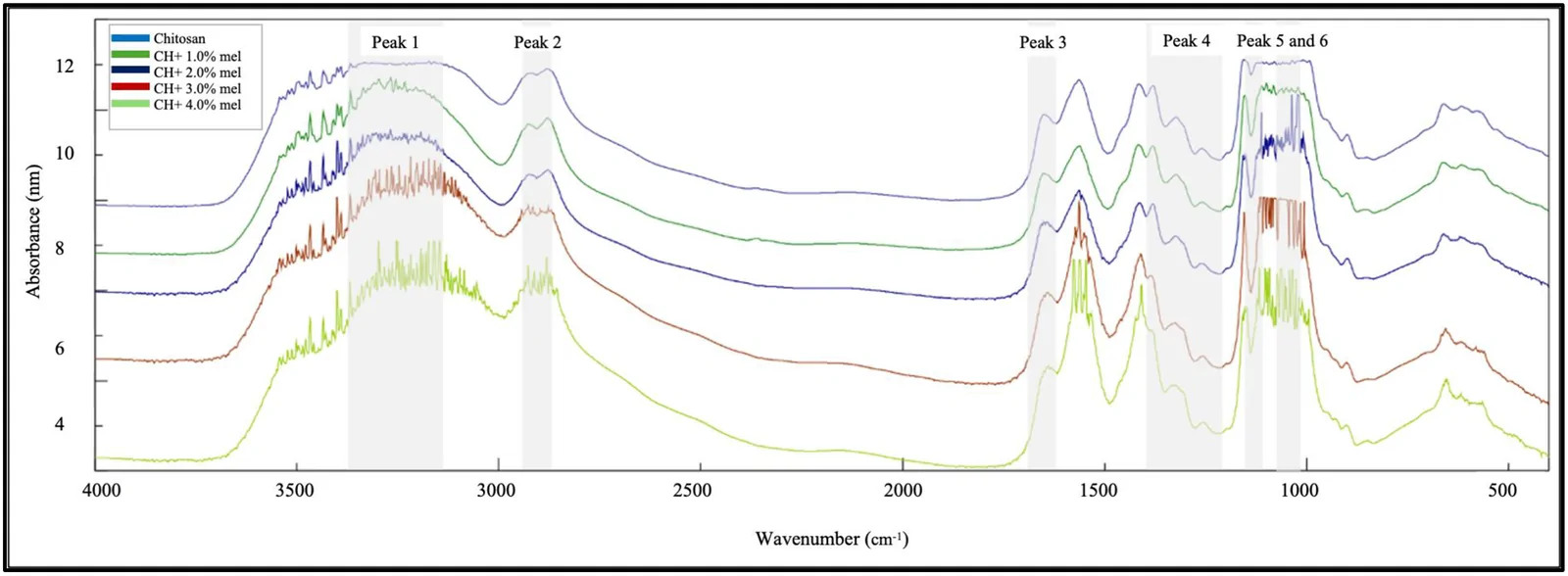
FT-IR spectra of the chitosan film as a control (light blue line) and of the chitosan films with the addition of 1.0% (dark green line), 2.0% (dark blue line), 3.0% (red line), or 4.0% microbial melanin (light green line).

#### 3.2.3 Colorimetric Analyses, UV-Shielding, and Optical Properties of the Films

The color of the surfaces of the chitosan films added with microbial melanin and the control film were analyzed, revealing a clear and progressive influence of the inserted melanin percentages on the optical responses (Figure 4). The λDOM values exhibited a gradual red shift from 492 nm of the control film to 495 nm value of the film containing 4.0% melanin, indicating a slight but consistent movement toward longer wavelengths as the pigment content increased. This behavior reflected the intrinsic absorption properties of melanin, which tends to darken the material and shift its chromatic response toward higher λDOM values. A similar concentration-dependent trend was observed in the CIE x–y chromaticity coordinates. The x-coordinate increased steadily from 0.408 (control) to 0.431 (4.0% melanin), whereas the y-coordinate decreased from 0.398 to 0.389 across the same concentration range. When represented in the CIE chromaticity diagram, the coordinates of the samples aligned along a well-defined trajectory, confirming a systematic shift within the color space with the increase of melanin percentages inserted in the chitosan films. Besides, this displacement suggested a progressive enhancement of the red–yellow component (higher x) combined with a slight reduction in the green contribution (lower y) of the colors of the films. Overall, the observed variations in λDOM and chromaticity coordinates demonstrated that melanin incorporation induced measurable and concentration-dependent modifications in the color of all films compared to the control. These changes were coherent with the broadband absorption profile of melanin and confirmed its effective integration within the polymer matrix. All these data were consistent with the UV–visible light shielding capabilities imparted by melanin to the chitosan films (Figure 5). The control film exhibited the highest transmittance across the entire spectrum, especially in the UV region (200–400 nm), indicating poor UV-blocking ability and high optical transparency. In comparison, all the chitosan films added with melanin showed reduced transmittance, particularly in the UV spectrum. This reduction was higher by increasing the melanin percentage, with the lowest transmittance value exhibited by the 4.0% melanin film, which had the strongest UV-shielding effect. Moving toward the visible region, transmittance values gradually increased for all samples compared to the values obtained in the UV region, although these values in all the melanin-incorporating films were always lower than the control one (Figure 5). The measurement of the absorbance of the films at 600 nm (Table 2), which increased from about 0.111 of the control to 0.201 of the 4.0% melanin chitosan-based film, allowed also to calculate the opacity of the samples, which increased from 0.002 to 0.004 ± 0.001, with a maximum reached when 4.0% of the microbial melanin was inserted.

**Figure 4:**
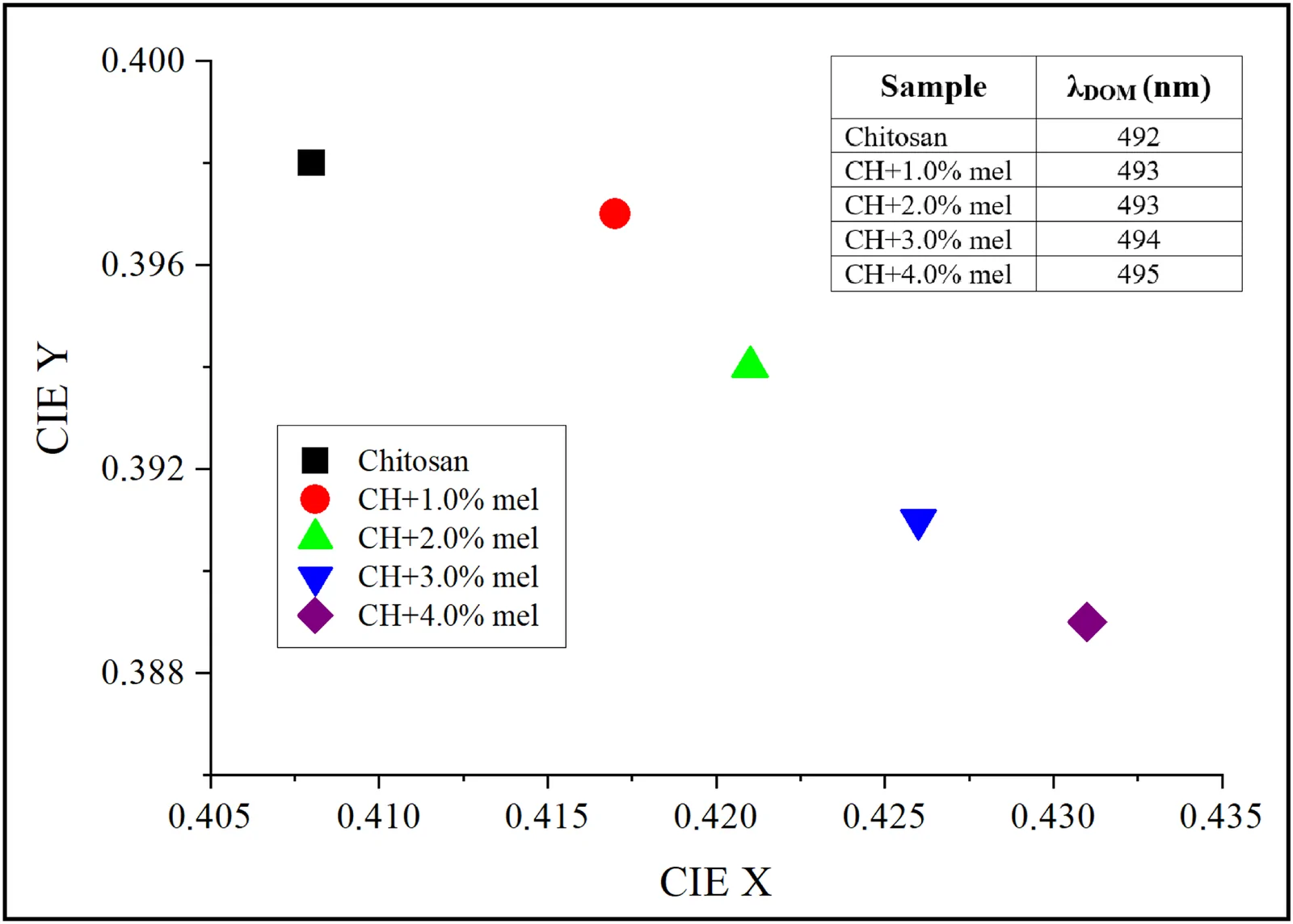
CIE x–y chromaticity plot of chitosan films added with different percentages of microbial melanin, in comparison with the control. The embedded table reports the dominant wavelength (λDOM) values for each film.

**Figure 5:**
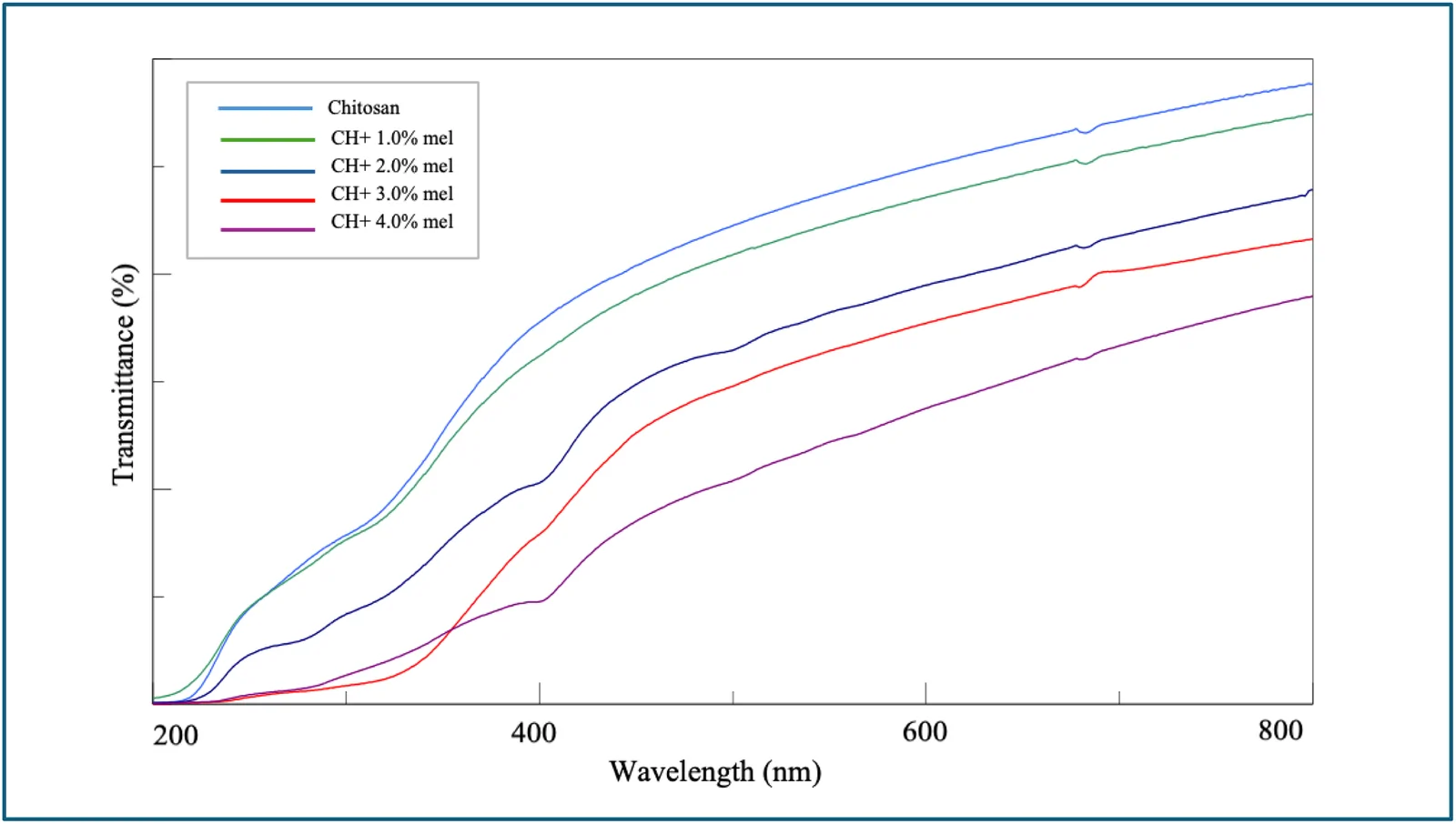
UV–visible transmittance (200–800 nm) of the chitosan film as control (black line) and of the chitosan films added with 1.0% (green line), 2.0% (blue line), 3.0% (pink line), or 4.0% (red line) microbial melanin.

**Table 2:** Absorbance Values and the Corresponding Calculated Opacity Values, of the Chitosan Films Added with Different Percentages of Microbial Melanin in Comparison with the Control^a^

	Abs_600 nm_	Opacity (Abs600/μm)
**Chitosan**	0.111 ± 0.001	0.002 ± 0.001^c^
Chitosan + 1.0% melanin	0.115 ± 0.001	0.002 ± 0.002^c^
Chitosan + 2.0% melanin	0.135 ± 0.001	0.002 ± 0.002^c^
Chitosan + 3.0% melanin	0.157 ± 0.001	0.003 ± 0.006^b^
Chitosan + 4.0% melanin	0.201 ± 0.001	0.004 ± 0.002^a^

a Data are presented as average values with standard deviations. Different letters within the same column indicate statistically significant differences (p < 0.05).

#### 3.2.4 Thermo-Gravimetric Analyses

To evaluate the effect of melanin on the structural properties of chitosan films, thermo-gravimetric analyses (TG) (in a nitrogen atmosphere) were also assessed. The TG spectra (Figure 6A) showed similar graph profiles with good thermal stability for all samples. Based on the three-stage thermal degradation profile, enhanced in derivative thermogravimetric (DTG) curves (Figure 6 B), the following temperature regions can be identified: a first mass-loss event (I) occurred between 30 and 100 °C (∼10% weight reduction), mainly attributed to the evaporation of physically adsorbed water. A second degradation step (II) was observed in the 100–220 °C range and can be associated with glycerol degradation.^11^ The main degradation process (III) was observed in the 200–380 °C temperature range due to chitosan backbone decomposition^21,22^ showing a prominent weight loss in all the samples. The decomposition temperature (T_d_) of the melanin-containing films underwent a small increase compared to neat chitosan, highlighting the potential of the pigment to increase the thermal stability of the hosting matrix. In particular, the control chitosan film exhibited a degradation temperature (T_d_) of 274 °C that was lower than that of the melanin-containing films. The T_d_ slightly increased to ∼281 and 286.14 °C for films containing 1.0 and 2.0% melanin, respectively. This behavior is likely attributable to strong intermolecular interactions between the amino groups of chitosan and the carboxylate groups of melanin, along with efficient dispersion and packing of melanin within the chitosan matrix.^28^ In addition, the films containing 3.0 and 4.0% melanin exhibited a T_d_ higher than the control but slightly lower than the previous values (284.94 and 283.75 °C, respectively), probably due to higher film heterogeneity, suggesting that above a certain melanin concentration threshold (2.0%) pigment particles might tend to form clusters.

**Figure 6:**
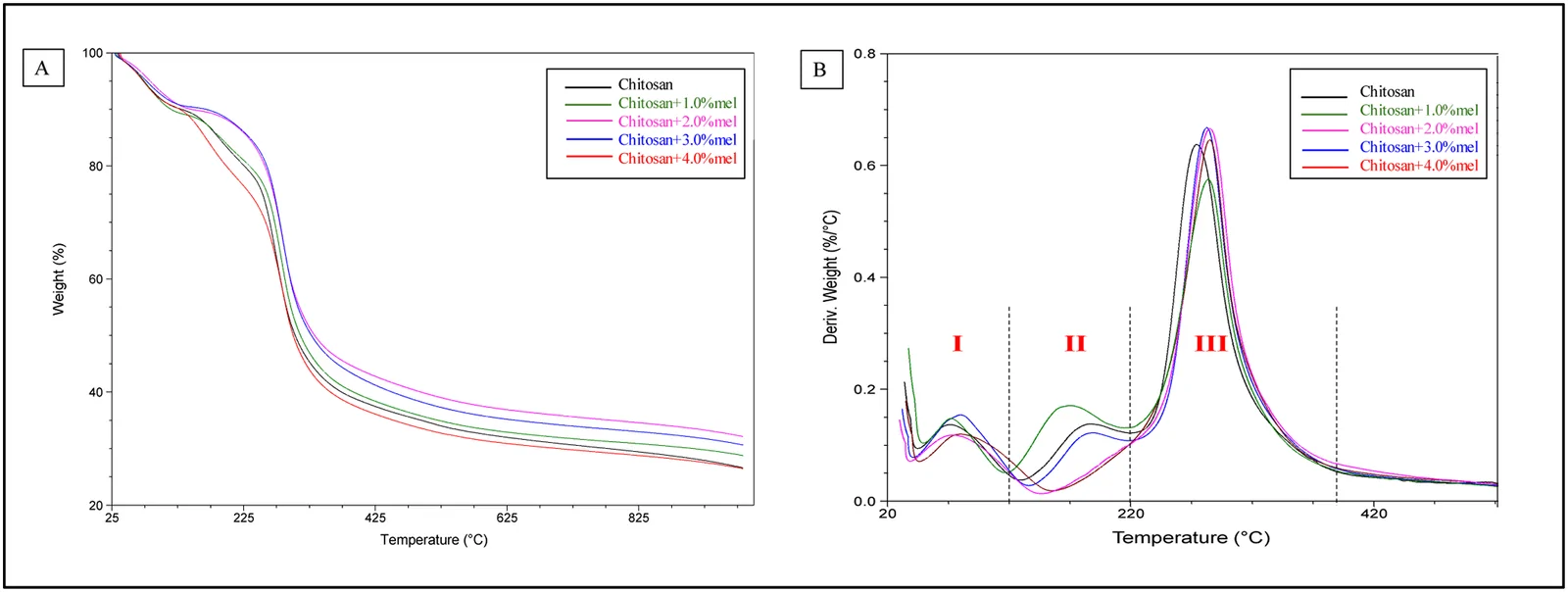
(**A**) TGA and (**B**) DTG curves of films: control chitosan and melanin-loaded films containing different melanin percentages.

#### 3.2.5 Thickness and Mechanical Properties

The thicknesses of all chitosan films added with melanin and of the control were measured (Table 3) and all showed values around 50.0 ± 1.0 μm. The mechanical properties such as tensile strength (TS) and elongation at break (EAB) were also determined, and they were found to improve with the increase of the inserted microbial melanin percentage (Table 3). As a matter of fact, the control films showed a TS of 26.1 ± 1.1 MPa and an EAB of 45.0 ± 4.0%, while when 4.0% melanin was added, the TS and EAB values of the films were significantly boosted up to 62.0 ± 2.3 MPa and 61.0 ± 4.8%, 2.4 and 1.3 times higher than the control, respectively.

**Table 3:** Thickness, tensile strength (TS), and elongation at reak (EAB) values of the films added with different percentages of microbial melanin, in comparison with the control[Table-fn tbl3fn1]

	Thickness (μm)	TS (MPa)	EAB (%)
**Chitosan**	50.0 ± 1.0^a^	26.1 ± 1.1^d^	45.0 ± 4.0^c^
Chitosan+ 1.0% melanin	49.0 ± 1.0^a^	31.0 ± 2.7^c^	50.0 ± 3.0^b, c^
Chitosan+ 2.0% melanin	51.0 ± 1.0^a^	34.0 ± 2.5^c^	55.0 ± 7.0^a, b^
Chitosan+ 3.0% melanin	49.0 ± 1.0 ^a^	58.0 ± 3.0^b^	59.0 ± 2.0^a^
Chitosan+ 4.0% melanin	50.0 ± 1.0^a^	62.0 ± 2.3^a^	61.0 ± 4.8^a^

i Data are presented as average values with standard deviations. Different letters within the same column indicate statistically significant differences (p < 0.05).

#### 3.2.6 Moisture Content, Water Solubility, and Swelling Ratio

Moisture content (MC), water solubility (WS), and swelling ratio (SR) of chitosan films added with different percentages of melanin exhibited consistent and concentration-dependent changes with increasing pigment content, compared to the control (Table 4). The MC of the chitosan film was 22.33 ± 0.48%, a value that decreased progressively up to 1.4-fold (15.57 ± 0.31%) when 4.0% microbial melanin was added. A similar reduction was also observed for the water solubility, which declined from 16.18 ± 1.90% in the control film to 11.07 ± 1.68% for the 4.0% melanin-chitosan-based film. In contrast, the swelling ratio showed an increasing trend, rising from 23.88 ± 0.24% of the control film to 28.39 ± 0.24% of the film with 4.0% melanin, with a boost of 1.3 folds

**Table 4:** Moisture Content (MC), Water Solubility (WS), and Swelling Ratio (SR) Percentages of the Different Chitosan Films Added with Different Percentages of Microbial Melanin in Comparison to the Control[Table-fn tbl4fn1]

	MC (%)	WS (%)	SR (%)
**Chitosan**	22.33 ± 0.48^a^	16.18 ± 1.90^a^	23.88 ± 0.24^c^
Chitosan+ 1.0% melanin	18.15 ± 1.76^b^	13.79 ± 2.02^b^	24.49 ± 0.93^c^
Chitosan+ 2.0% melanin	17.55 ± 0.11^b^	12.38 ± 0.70^b^	26.07 ± 0.18^b, c^
Chitosan+ 3.0% melanin	16.62 ± 1.66^c^	11.87 ± 2.35^b^	27.21 ± 0.22^a, b^
Chitosan+ 4.0% melanin	15.57 ± 0.31^c^	11.07 ± 1.68^b^	28.39 ± 0.24^a^

i Data are presented as average values with standard deviations. Different letters within the same column indicate statistically significant differences (p < 0.05).

#### 3.2.7 Gas Permeability

The CO_2_ permeability of the control film was 0.063 cm^3^·mm·m^–2^·d^–1^·kPa^–1^, while after the addition of microbial melanin, this value progressively decreased with increasing pigment percentages, by up to 2.2-fold (0.033 cm^3^·mm·m^–2^·d^–1^·kPa^–1^) (Table 5). In terms of water vapor permeability (WVP), the chitosan film showed a value of 2.30 g·mm·m^–2^·d^–1^·kPa^–1^, similar to the 1.0% melanin-added film (Table 5), while by inserting microbial melanin percentages a decrease in WVP was observed up to 1.9-fold.

**Table 5:** Permeability to CO_2_ and Water Vapor of the Different Chitosan Films Added with Different Percentages of Microbial Melanin in Comparison to the Control[Table-fn tbl5fn1]

	CO_2_ (cm^3^ mm m^–2^ d^–1^ kPa^–1^)	WVP (g mm m^–2^ d^–1^ kPa^–1^)
**Chitosan**	0.063 ± 0.008^a^	2.30 ± 0.91^a^
Chitosan+1.0% melanin	0.046 ± 0.001^b^	2.28 ± 0.80^a^
Chitosan+2.0% melanin	0.042 ± 0.009^b, c^	1.91 ± 0.23^b^
Chitosan+3.0% melanin	0.039 ± 0.010^c^	1.32 ± 0.90^c^
Chitosan+4.0% melanin	0.033 ± 0.002^d^	1.19 ± 0.60^c^

i Data are presented as average values with standard deviations. Different letters within the same column indicate statistically significant differences (p < 0.05).

#### 3.2.8 Water Contact Angle

The chitosan film (control) exhibited a contact angle of 100.67 ± 1.72, indicative of a relatively hydrophobic surface; however, the addition of increasing percentages of melanin caused a progressive decrease of the contact angle values, suggesting an increment of the film surface hydrophilicity (Figure 7). Specifically, the contact angle progressively reduced to 99.64 ± 2.75, 88.36 ± 2.75, 84.63 ± 1.29, and 81.59 ± 1.70 with 1.0, 2.0, 3.0, and 4.0% melanin addition, respectively, with a maximum reduction of 1.28-fold (Figure 7). This reduction reflects increased surface wettability due to the presence of polar catechol, quinone, and indolic groups of the eumelanin structure, which increase surface free energy and promote water spreading.

**Figure 7:**
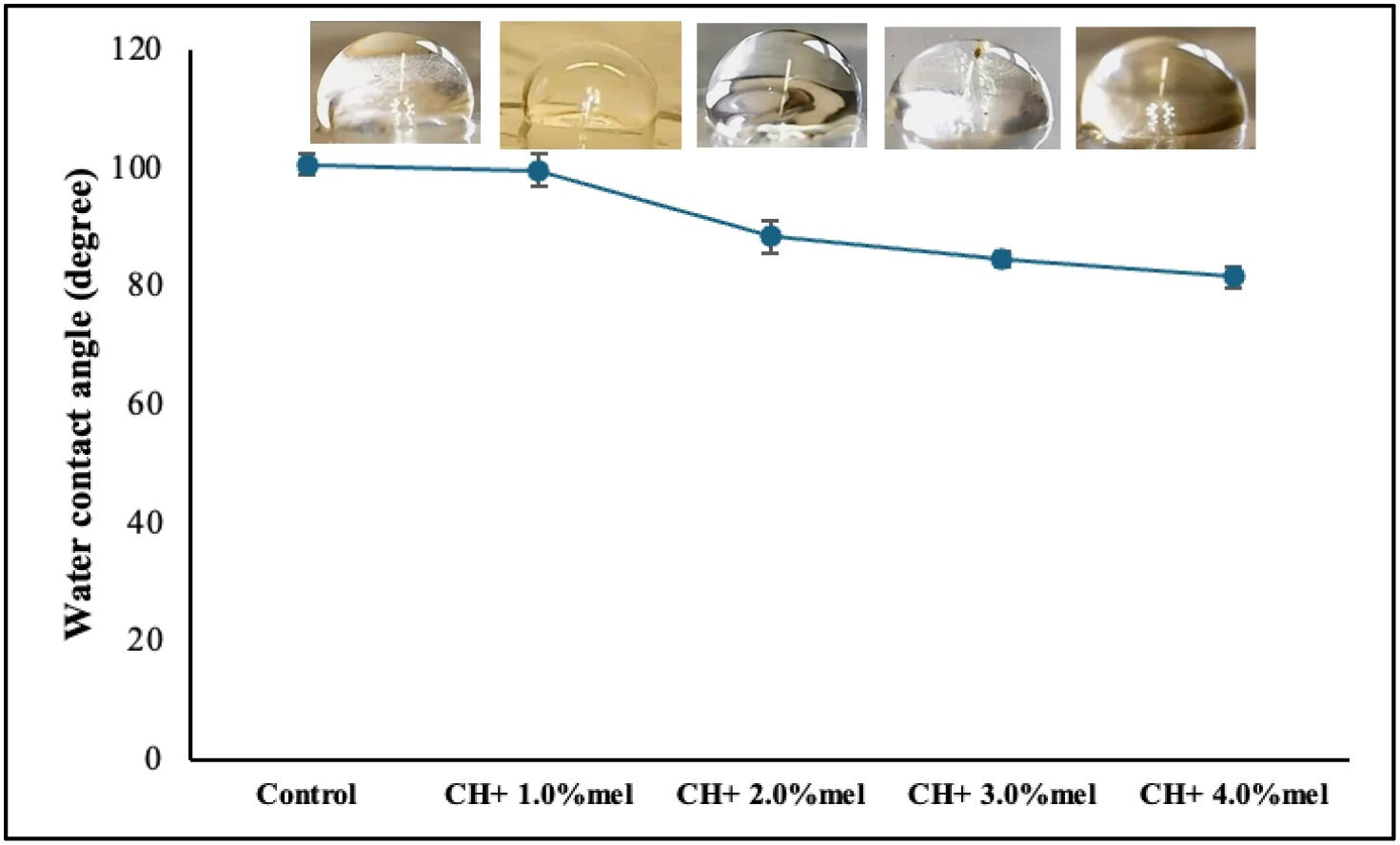
Water contact angle of the chitosan films as control and of the chitosan films with 1.0%, 2.0%, 3.0%, and 4.0% of inserted microbial melanin.

#### 3.2.9 Antioxidant Activity

The ferric reducing antioxidant power (FRAP) assay evaluates antioxidant capacity based on the ability of compounds to act as electron donors, reducing ferric (Fe^3+^) to ferrous (Fe^2+^) ions. So, by adding melanin in the films, the FRAP values increased significantly in a dose-dependent manner, reaching up to 88.34 ± 0.02 μM Fe^2+^ equivalents at 4.0% (w/w) melanin, compared to the control, which exhibited a reducing power of 6.09 ± 0.02 μM Fe^2+^ equivalents (Figure 8A). This enhancement reflected the strong electron-donating capacity of melanin’s indole and quinone groups and demonstrated that increasing the melanin percentage in the films effectively boosted the redox potential of the chitosan matrix, clearly suggesting that melanin acts as an antioxidant component capable of enhancing the functional properties of chitosan-based films. The free radical scavenging activity of chitosan-based films was also determined by DPPH (Figure 8B). The incorporation of microbial melanin into chitosan films resulted in a statistically significant enhancement of DPPH scavenging activity compared to the control film, which exhibited an inhibition value of 16.74%, which increases with melanin percentages (from an inhibition of about 20.74% to 49.22%), moving from 1.0 to 4.0%.

**Figure 8:**
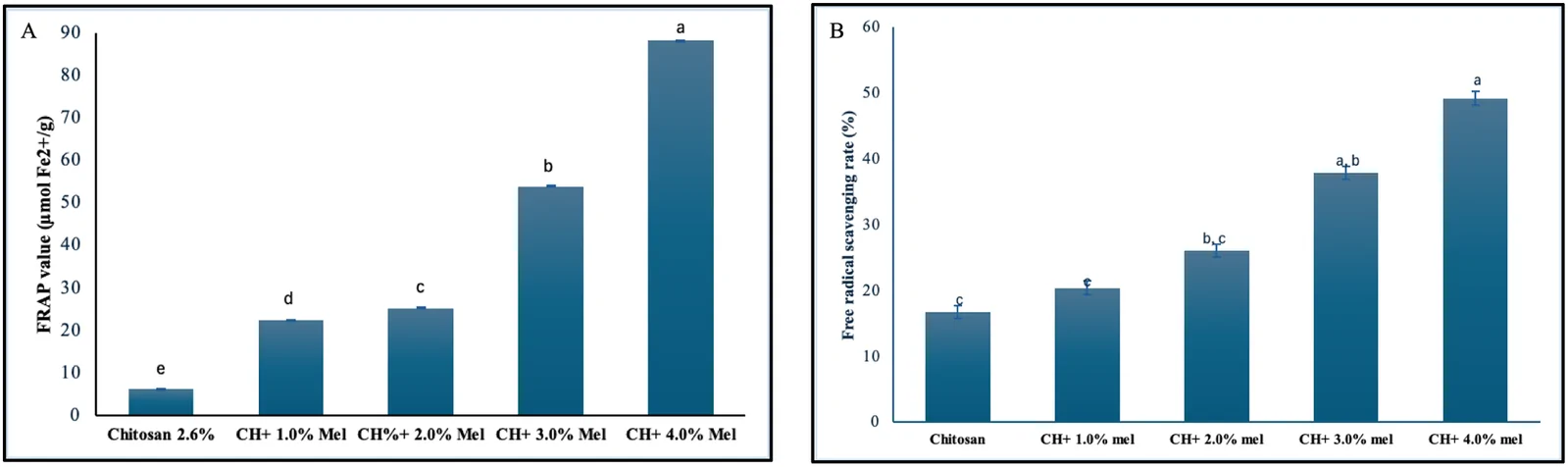
Antioxidant activity of film extracts evaluated by (A) ferric reducing antioxidant power assay (FRAP) and (B) DPPH scavenging activity assay. Different letters within the same column indicate significant differences (*p* < 0.05).

#### 3.2.10 Antimicrobial Activity

Liquid microbial cultures of *P. aeruginosa* and *E. faecalis* (5 × 10^5^ CFU•mL^–1^) were added to the FFSs of 4.0% melanin and of the control to evaluate their antimicrobial activity. For *E. faecalis*, the control FFS reduced the viable cell count by approximately 1.7 ± 0.3 log units after 24 h compared to the initial bacterial load, confirming a slow antimicrobial chitosan activity.^28,29^ Instead, the FFS containing 4.0% melanin completely inhibited bacterial growth after 24 h. This clear, stronger inhibitory effect suggested that the microbial melanin effectively contributed to the antibacterial performance against *E. faecalis*. In the case of *P. aeruginosa*, complete growth inhibition was observed after 24 h for both FFSs, including the control formulation. These results demonstrated that the developed chitosan-based formulations containing microbial melanin exhibited a clear microorganism-dependent antibacterial activity, producing strong inhibition against *E. faecalis*, whereas the control formulation was already sufficient to completely inhibit *P. aeruginosa*.

## 4 Discussion

Manufacturing of chitosan materials has been widely explored over the last decades, and numerous bioactive molecules have been inserted in them to enhance or add specific properties, but so far in the literature very few papers have reported the addition of melanin. The numerous physical–chemical and biological properties of melanin make it an ideal biomolecule for insertion in new biodegradable films and materials,^12^ but the difficulties and the high costs of recovering and purification of this pigment from animal sources or producing it by chemical synthesis have limited its application.^13^ Although, in the literature, the melanin used to manufacture chitosan-based materials was mainly of animal origin or of chemical synthesis while the employment of bacterial melanin has not been reported so far. Besides, this pigment has always been used in chitosan materials as nanoparticles and not as dispersed form.^10,11^ For example, synthetic melanin-like nanoparticles were prepared to reinforce chitosan nanocomposite films potentially useful for active food packaging and biomedical applications, while, in another case, epigallocatechin-3-gallate (EGCG)-loaded melanin-like nanoparticles (EGCG@MNPs) were incorporated into a chitosan matrix to prepare active nanocomposites for food packaging films with enhanced antibacterial and antioxidant properties.^11,21^ Melanin from cuttlefishes was used, instead, in the preparation of composite nanoparticles (melanin-glycine-C60 nanoparticles) to be inserted in 3D chitosan hydrogels for wound healing.^29^ The dispersed form of animal-origin melanin was, instead, recently used in the manufacturing of alginate films prepared by ion gelation, also using glycerol (30.0%) as a plasticizer.^30^ More recently, the improvements obtained in the biotechnological production process of microbial melanin made this approach a valid alternative to other manufacturing processes of the pigment, and a more reliable and sustainable route of manufacturing. Both bacterial and fungal species might produce melanin, but some bacteria have been shown to produce the pigment more easily and quickly in liquid media in runs of 96–120 h, and as an extracellular product, so its recovery and purification are simple. Instead, the melanin synthesized by fungi is generally produced via solid-state fermentation, in longer runs of at least 168 h^13^, and, as an intracellular product, is therefore more difficult to be purified, also because it must be extracted. Some *Streptomyces* strains have been recently employed as microbial cell factories to produce bacterial melanin.^13–17^ In particular, in previous papers, the pigment produced on a glucose, yeast, and malt extract medium by *Streptomyces nashvillensis* DSM 40314, and then purified, was demonstrated to have a eumelanin-like structure, thus similar to mammalian melanin, to have high resistance to UV–visible light (>80% for 6 h of exposure), high thermal stability (from about 80.4 to 53.0% when moving from 40 to 100 °C), clear redox activity, moderate antioxidant activity, and antimicrobial action against *E. faecalis* ATCC 29212 and *E. faecium* DSMZ 17050.^13,17^ In this paper, this bacterial melanin was dispersed, for the first time, in chitosan film-forming solutions containing glycerol as a plasticizer (30.0%, a percentage similar to that reported before by Verduzco-Grajeda et al., 2024,^30^ after optimization of the dissolution conditions, such as the pH value, in order to obtain complete chitosan solubilization and melanin dispersion. The obtained FFSs were used to manufacture films, by the casting method, employing a coat-master instrument, after adding different percentages of microbial melanin. As expected, the insertion of the pigment in the chitosan films was mainly driven by ionic interactions, as shown by FT-IR analyses, producing a very homogeneous structure in which the pigment was totally dispersed, and whose superficial roughness increased by increasing melanin percentages, similarly to what was already reported by SEM analyses for alginate films containing dispersed melanin of animal origin.^30^ Taking in consideration the properties of melanin, it was not surprising to note that an enhancement of the color of the films was obtained by increasing the percentages of the inserted melanin, as well as an enhanced shielding effect, and thus a decrease in the UV–visible light transmittance that perfectly correlated with the increase of opacity values. This behavior might be easily attributed to melanin UV and visible-light protecting properties, due to its complex conjugated aromatic structure,^31^ but it is also in line with the increased shielding behaviors and optical properties of chitosan films reinforced with synthetic melanin-like nanoparticles or with EGCG-loaded melanin nanocomposites, as previously reported.^11,21^ All these data might also suggest exploring in the future the possible use of chitosan-melanin films as effective biobased UV-shielding materials in food packaging.^32^ Instead, the insertion of melanin in the chitosan-based films did not significantly change the thermal properties, which remained stable and similar to the control. These results agree with what was already reported for synthetic melanin nanoparticles inserted in chitosan nanocomposite films,^10^ as well as in polylactic acid or in agar composite films.^33,34^ On the other hand, the dispersion of melanin, as a powder, within the chitosan matrix clearly increased the mechanical properties of the films, in agreement with previous data that demonstrated that the pigment in the form of nanoparticles enhanced the mechanical integrity of manufactured materials.^10,11^ The improved tensile properties might be attributed to the dispersion of melanin throughout the polymer network, but the pigment addition may also restrict chain mobility in a controlled manner, contributing to increased elongation at break, and acting as a moderate plasticizer enhancing the polymer chain extensibility. The enhancement of the mechanical behaviors of the films might be attributed to the capacity of melanin to form both hydrogen bonds and ionic interactions with the chitosan polymer chains, thanks to its numerous hydrophilic functional groups (hydroxyl, carboxyl, amine groups), thus creating a more cohesive and interactive matrix, resulting in enhanced stress transfer under load. The hydrophilic functional groups of the microbial melanin not only allowed the pigment to act as a natural reinforcing agent in chitosan films, but also as surface-modifying agent, enhancing the surface hydrophilicity of the films at the increase of melanin percentages, creating a structure that help the water spreading. But melanin provided also a partially hydrophobic character to the film matrix, limiting its ability to retain and to dissolve in water as a decreasing moisture content and water solubility values were noted. These results agree to the data reported before for melanin nanoparticles in chitosan films^26^ and for the whey protein-melanin films, in which the pigment addition reduced the solubility of approximately 13.0–21.0%. Also, the swelling ratio slightly increased, allowing higher structural flexibility and expansion of the films when hydrated. Similar behavior was also reported for melanin-incorporating whey protein films, in which the swelling ratio increased despite the reduced solubility.^33^ These results might be attributed to the insertion of melanin phenolic rings within the chitosan polymers, which partially disrupt the tight packing due to the hydrophilic interactions, thus promoting a partially opened, water-accessible network. It seemed that melanin acted as a network modifier in the chitosan films, decreasing solubility while improving the water-responsive expansion behavior, thanks to its hydrophilic and hydrophobic functional groups; the balance between the interactions of these groups with the chitosan polymer chains might also be responsible for the diminishing of CO_2_ and water vapor permeability by changing the tightness of the matrix, producing a less permeable structure and thereby limiting gas diffusion. Similar effects were observed in other polysaccharide-based films modified with melanin-like nanoparticles^26^ as hydrophobic additives might increase tortuosity and reduce water vapor diffusion.^35^ Furthermore, despite the observed increase in surface hydrophilicity of the melanin-added films, water vapor permeability (WVP) decreased progressively with increasing melanin content. This contradiction arises from the distinction between surface thermodynamics and bulk transport phenomena. The contact angle reflects interfacial energy at the solid–liquid boundary, whereas WVP is governed by diffusion through the polymer matrix. The incorporation of microbial melanin promotes hydrogen bonding and π–cation interactions with chitosan, leading to network densification, reduced free volume, and increased diffusion tortuosity. As reported for hydrogen-bonded polysaccharide systems, internal matrix cohesion can dominate vapor transport behavior even when surface polarity is enhanced.^2,6^ Consequently, water spreading at the surface is facilitated, while bulk vapor transport is hindered. The increased antioxidant activity of the films and antimicrobial action were clearly connected with the unique structure of the pigment, which provided new specific properties to the chitosan films. All these results demonstrate that, in future, the manufactured melanin-based chitosan films might be suitable for different applications.

## 5 Conclusions

For the first time, microbial melanin biotechnologically produced and purified by *Streptomyces nashvillensis* DSM 40314 was inserted and dispersed at different percentages in FFSs of chitosan to obtain films by the casting method. The incorporation of biotechnologically produced microbial melanin into chitosan films resulted in concentration-dependent structural and functional modifications, demonstrating that melanin behaves as an active modifier. This work established for the first time that microbial melanin can act as a multifunctional bioactive molecule capable of enhancing mechanical integrity, barrier performance, antioxidant activity, and UV-shielding properties in chitosan-based materials through noncovalent bonds.

## Data Availability

The data presented in this study are available from the corresponding author upon reasonable request.
